# SARS-CoV-2 mutations acquired during serial passage in human cell lines are consistent with several of those found in recent natural SARS-CoV-2 variants

**DOI:** 10.1016/j.csbj.2022.04.022

**Published:** 2022-04-21

**Authors:** Hoyin Chung, Ji Yeong Noh, Bon-Sang Koo, Jung Joo Hong, Hye Kwon Kim

**Affiliations:** a Department of Biological Sciences and Biotechnology, College of Natural Sciences, Chungbuk National University, Cheongju 28644, Republic of Korea; b National Primate Research Center, Korea Research Institute of Bioscience and Biotechnology, Cheongju, Republic of Korea

**Keywords:** SARS-CoV-2, Variant, Mutation, SNP, Evolution

## Abstract

Since the outbreak of coronavirus disease (COVID-19) in 2019, severe acute respiratory syndrome coronavirus 2 (SARS-CoV-2) has evolved into diverse variants. Here, an early isolate of SARS-CoV-2 was serially passaged in multiple cell lines of human origin in triplicate, and selected mutations were compared to those found in natural SARS-CoV-2 variants. In the spike protein, Q493R and Q498R substitutions from passaged viruses were consistent with those in the B.1.1.529 (Omicron) variant. Y144del and H655Y substitutions from passaged viruses were also reported in B.1.1.7 (Alpha), P.1 (Gamma), and B.1.1.529 (Omicron) variants. Several single nucleotide polymorphisms (SNPs) found in first-passaged viruses have also been identified as selected mutation sites in serially passaged viruses. Considering the consistent mutations found between serially passaged SARS-CoV-2 and natural variants, there may be host-specific selective mutation patterns of viral evolution in humans. Additional studies on the selective mutations in SARS-CoV-2 experiencing diverse host environments will help elucidate the direction of SARS-CoV-2 evolution.

**Importance:**

SARS-CoV-2 isolate (SARS-CoV-2/human/KOR/KCDC03-NCCP43326/2020) was serially passaged in A549, CaCO2, and HRT-18 cells in triplicate. After 12 times of serial passages in each cell lines, several consistent selected mutations were found on spike protein between the serially passaged SARS-CoV-2 in human cell lines and recent natural variants of SARS-CoV-2 like omicron. On the non-spike protein genes, selected mutations were more frequent in viruses passaged in Caco-2 and HRT-18 cells (Colon epithelial-like) than in those passaged in A549 cells (Lung epithelial-like). In addition, several SNPs identified after one round of passaging were consistently identified as the selected mutation sites in serially passaged viruses. Thus, mutation patterns of SARS-CoV-2 in certain host environments may provide researchers information to understand and predict future SARS-CoV-2 variants.

## Graphical abstract

**Figure:**
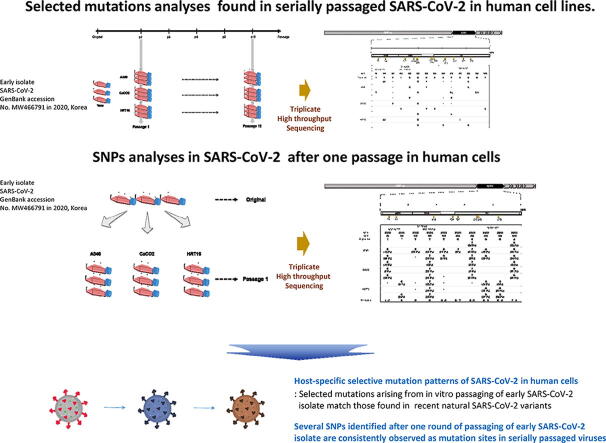

## 1 Introduction

Since the outbreak of coronavirus disease (COVID-19) in 2019, severe acute respiratory syndrome coronavirus 2 (SARS-CoV-2), has spread worldwide. The origin of SARS-CoV-2 is not yet fully understood. Among possible zoonotic sources, the RATG13 bat coronavirus strain from the horseshoe bat (*Rhinolophus affinis*) shares 96.2% sequence identity with SARS-CoV-2 [1]. In addition, coronaviruses sharing 85.5–92.4% sequence similarity with SARS-CoV-2 were identified in Malayan pangolin (*Manis javanica*) [2]. However, a SARS-CoV-2-specific feature, the polybasic cleavage site at the junction of S1 and S2 of the spike protein, is not evident in these viruses [3]. Considering the similarity of the SARS-CoV-2-related viruses found in these animals, it is likely that a closely related natural host of SARS-CoV-2 was infected from the intermediate horseshoe bat and/or Malayan pangolin hosts and has not yet been discovered. This additional host would allow SARS-CoV-2-specific variants not seen in the intermediate hosts to arise [4].

SARS-CoV-2 has continuously evolved into diverse variants with approximately 0.035 amino acid substitutions occurring per day [5]. Genomic mutation analysis showed that SARS-CoV-2 variants clustered into nine clades, with clade G and its derivatives becoming rapidly dominant around the world [6]. Some variants have received attention for their potential impact on infectivity and pathogenicity. For example, the D614G and P681R mutations in the spike protein were associated with increased viral replication in human lung epithelial cells [7] and enhanced viral pathogenicity [8], respectively. Notably, some mutations, such as N439K and E484K, are thought to be related to protective immune responses [9], [10], [11]. The various lineages of SARS-CoV-2 variants have been classified into four groups: variant being monitored (VBM), variant of interest (VOI), variant of concern (VOC), and variant of high consequence (VOHC) [12]. Delta (B.1.617.2 and AY lineages) and Omicron (B.1.1.529) were listed as VOCs, while others were included in the VBM category.

The evolution of SARS-CoV-2 is ongoing, and further progression is inevitable due to the ability of viruses to frequently mutate. Most viral mutations are deleterious, but a relatively large population of variants may allow interhost transmission to overcome a wide transmission bottleneck [13], [14]. In addition, considering the quasispecies nature of SARS-CoV-2, population-based analysis is necessary to understand viral evolution [15].

In general, transmission bottlenecks differ based on viral infection cycles and host dynamics. As viruses are obligate parasites that need host cells for their replication, the host cell environment is an essential factor impacting their evolution. In this regard, hosts can affect the natural selection of the next generation of viruses from quasispecies. Here, SARS-CoV-2 evolution patterns in different host cells were monitored using high-throughput sequencing. Additionally, the mutations arising from infection of each host cell type was compared with natural mutations found in currently circulating SARS-CoV-2 variants.

## 2 Results

### 2.1 SARS-CoV-2 replication characteristics in A549, Caco-2, and HRT-18 cells

**Fig. 1:**
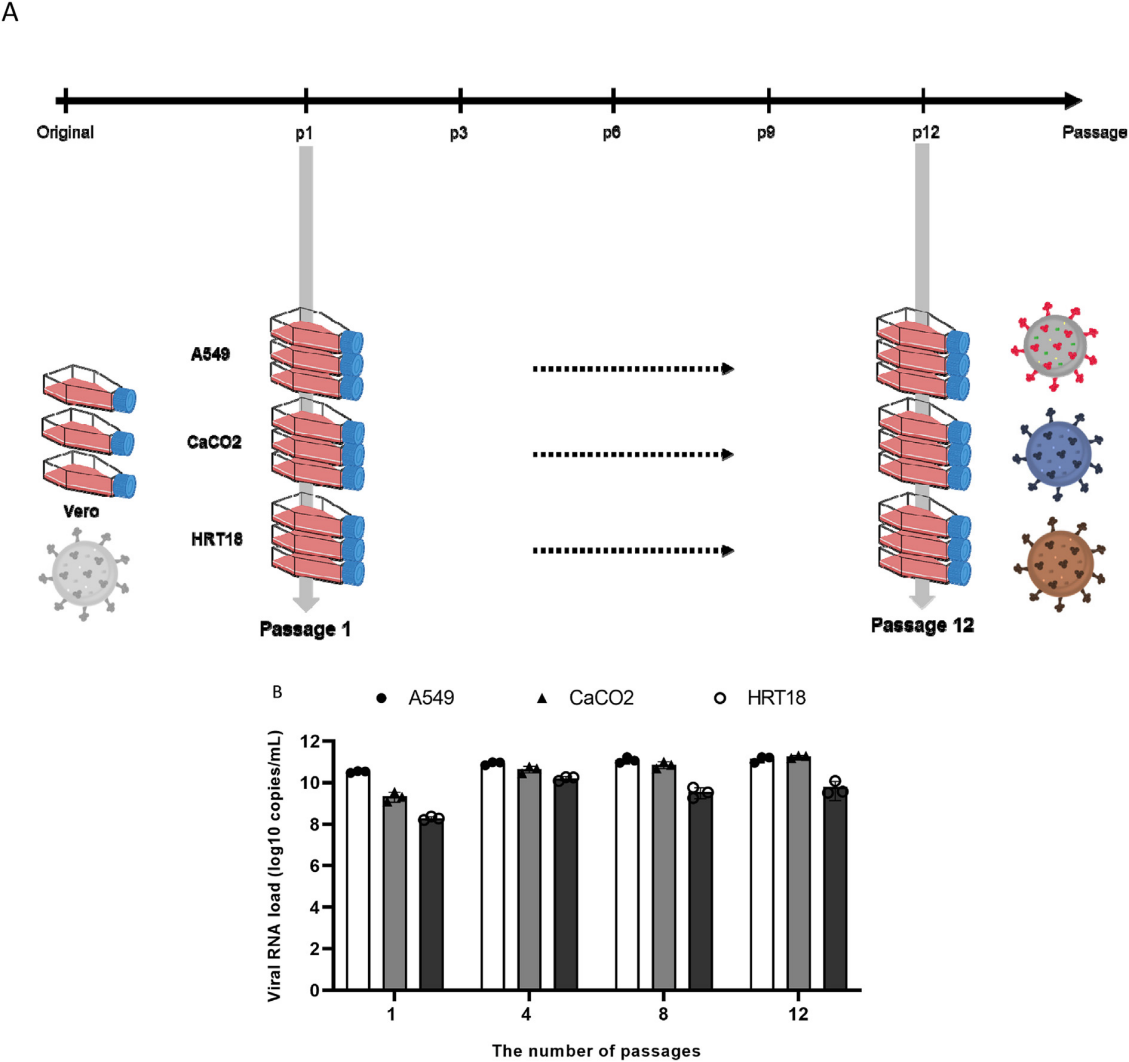
SARS-CoV-2 serial passage scheme in A549, Caco-2, and HRT-18 cell lines. A, The original SARS-CoV-2 isolate was passaged in triplicate in each cell line. Samples from passage 1 and 12 were analyzed with high-throughput sequencing. B, Real-time RT-PCR results from SARS-CoV-2 serially passaged in A549, Caco-2, and HRT-18 cell lines. RNA extracted from passages 1, 4, 8, and 12 was tested with real-time RT-PCR targeting the ORF1b region of the virus^28^. The viral RNAs were quantified using a standard curve obtained from the SARS-CoV-2 RNA standard (Twist Synthetic SARS-CoV-2 RNA Control MT007544.1, Twist Bioscience, USA).

First, the ability of SARS-CoV-2 to replicate in a variety of cell lines was tested. An early SARS-CoV-2 isolate (SARS-CoV-2/human/KOR/KCDC03-NCCP43326/2020, GenBank accession No. MW466791) was passaged four times in Vero cells and subsequently used as the original virus for serial passage in A549, Caco-2, and HRT-18 cells, which were cell lines of human origin (Fig. 1A). Throughout the passages, viral RNA was quantified by real-time RT-PCR. It was found that SARS-CoV-2 was able to continuously replicate in A549, Caco-2, and HRT-18 cells during serial passage. Viral RNA copies were maintained at more than 10 log_10_ RNA copies/ml in passages 1, 4, 8, and 12 in A549 cells (Fig. 1B). In Caco-2 cells, viral RNA copies from the first passage were relatively low at 9.3 log_10_ RNA copies/ml, but they gradually increased as the virus was passaged, with 10.6, 10.8, and 11.3 log_10_ RNA copies/ml in passages 4, 8, and 12, respectively. Viral RNA copies were lower in HRT-18 cells than in A549 and Caco-2 cells, with 8.3, 10.2, 9.5, and 9.7 log_10_ RNA copies/ml at passages 1, 4, 8, and 12.

### 2.2 Sequence data

On average, 56,543,462 reads per sample were obtained by high-throughput sequencing. After quality control, an average of 56,542,766 trimmed reads per sample were used for assembly and mapping (supplement table 2). Reads were mapped to the reference genome of SARS-CoV-2 and to the original viruses with a mean 95.7% of reads with phred score (>Q30). SARS-CoV-2 genomic sequences and mutations were analyzed using an average of 15,749,985 mapped reads, with an average confidence mean of 1,738,167.4 per sample (supplement table 2). All sequence data have been deposited in the GenBank Sequence Read Archive (BioProject number PRJNA786802).

### 2.3 Selected spike protein mutations from consensus genomic sequences of passaged SARS-CoV-2

**Fig. 2:**
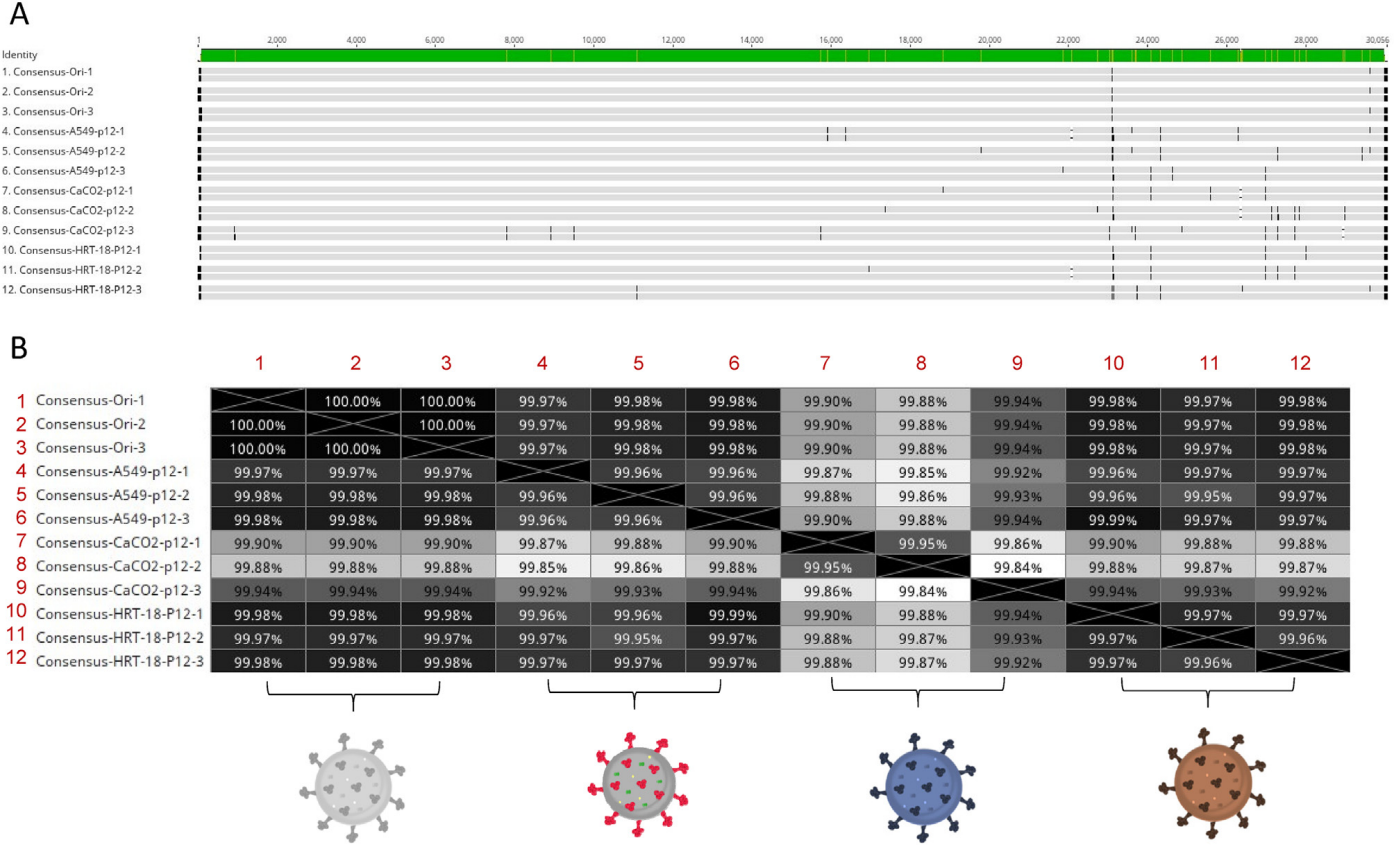
Comparison of consensus genomic sequences of original and serially passaged SARS-CoV-2 isolates in human cell lines. A, multiple sequence alignment of consensus genomic sequences created using MAFFT v7.450 integrated in Geneious Prime 2021.2.2. B, Consensus genomic sequences of original and passaged SARS-CoV-2 in A549, Caco-2, and HRT-18 cell lines.

Three consensus genomic sequences were constructed from deep sequencing of the original SARS-CoV-2 isolates used for inoculation into A549, Caco-2, and HRT-18 cells. The three genomes had 100% sequence similarity, indicating that the starting viral genome was identical in each cell type. The consensus genomic sequences of SARS-CoV-2 passaged in each cell type had some mutations and deletions that were sporadic and cell-type specific, and some that were conserved between all cell types compared to the original SARS-CoV-2 isolate (Fig. 2a). The sequence similarities of passaged SARS-CoV-2 compared to the original isolate were 99.97–99.8% (A549 cells), 99.88–99.94% (Caco-2 cells), and 99.97–99.98% (HRT-18 cells) (Fig. 2b).

**Fig. 3:**
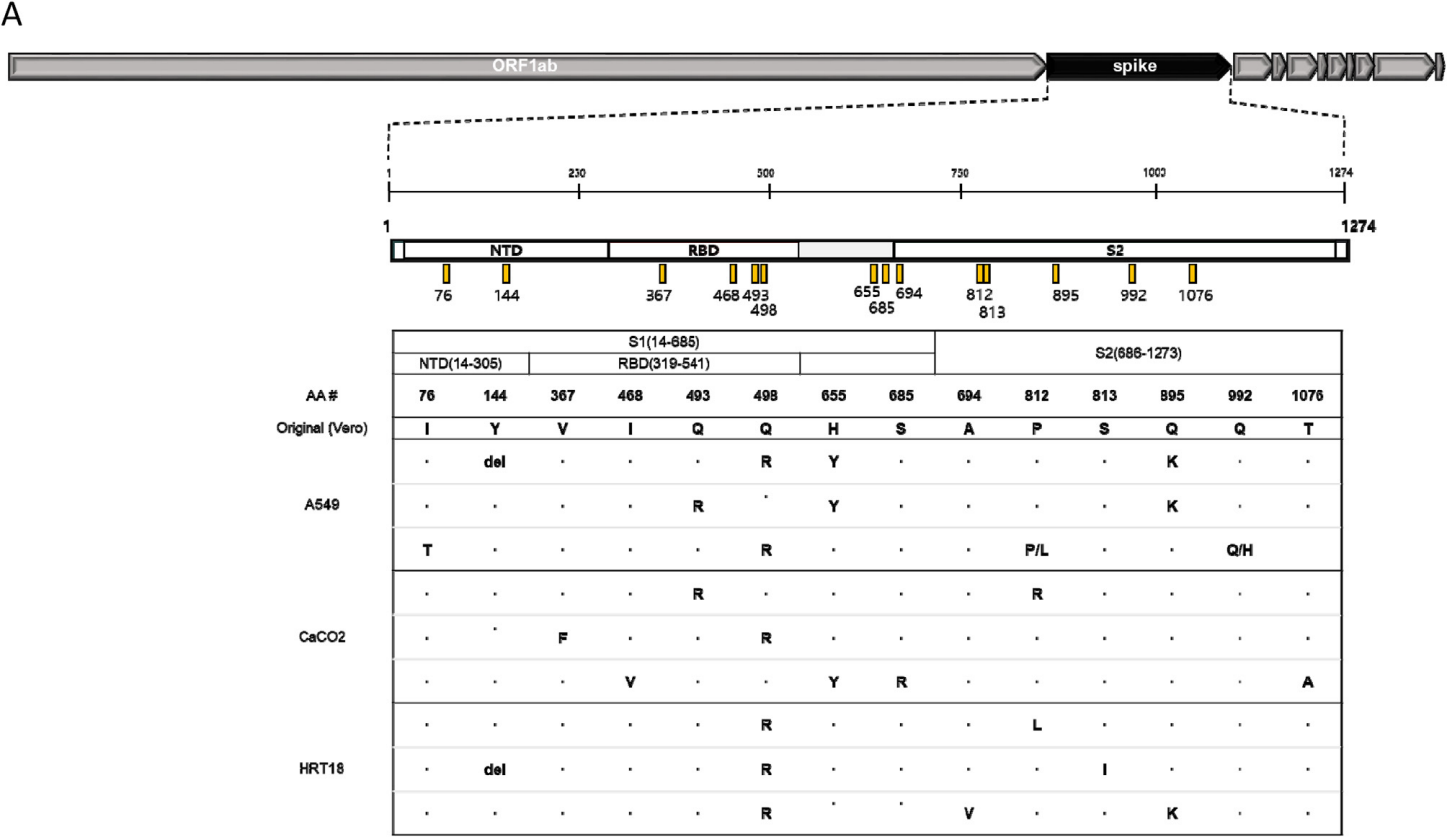

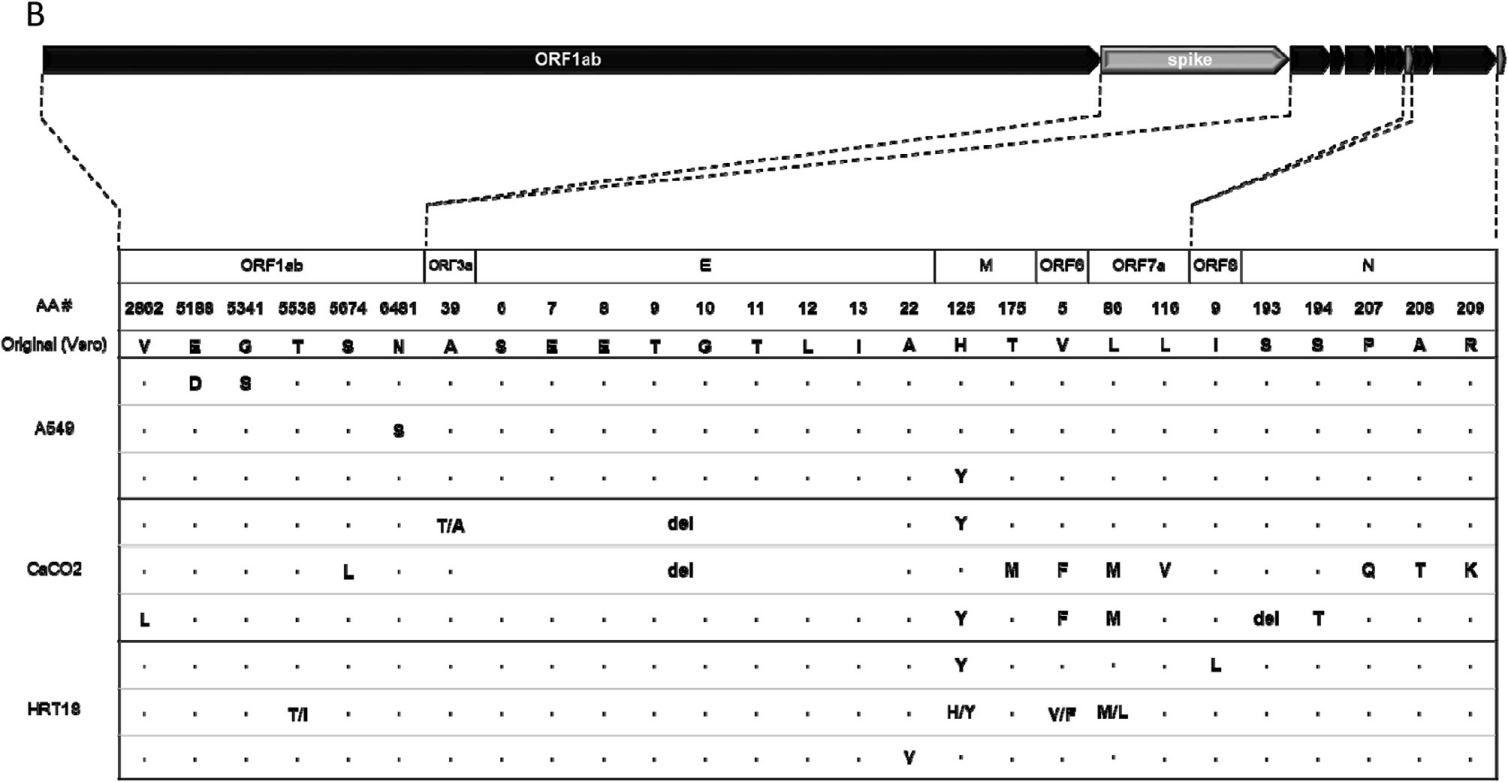
Selected mutations found in serially passaged SARS-CoV-2 in human cell lines. Amino acid sequences of SARS-CoV-2 genes were compared between the original and passaged viruses. A, amino acid substitutions on the spike protein. B, amino acid substitutions in non-spike protein peptides; del, deletion of amino acid.

In total, 14 selected mutation sites were identified in the spike proteins of passaged viruses (Fig. 3a). In addition, E484D mutations were observed in all viruses, including the original strain. Q498R in the receptor-binding domain (RBD) is a common mutation found in most circulating strains of the virus. It was found that all viruses passaged in HRT-18 cells, two of the three viruses passaged in A549 cells, and one of the three viruses passaged in Caco-2 cells had the Q498R mutation. An additional Q493R mutation was found in one virus each passaged in A549 and Caco-2 cells. Y144del mutations in the N-terminal domain (NTD) were found in one virus each passaged in A549 and HRT-18 cells. Two viruses in A549 cells and one virus in Caco-2 cells had H655Y mutations. There were Q895K mutations in the S2 domain of two viruses passaged in A549 cells and one virus passaged in HRT-18 cells. At position 812 in the S2 domain, either P812L or P812R mutations were found in one of the three viruses passaged in all cell types. There were also several sporadic mutations (I76T, V367F, I468V, S685R, A694V, S813I, Q992H, and T1076A) in one of the viruses analyzed in this study.

### 2.4 Selected non-spike protein mutations from passaged SARS-CoV-2

The ORF1ab gene was translated using a slippery sequence, UUUAAACGGG, and selected mutations were analyzed (Fig. 3b). Although several sporadic mutations were found (V1862L, E5188D, G5341S, T5538I, S5674L, and N6481S), they were not consistently associated. The mutations observed in the translated amino acid sequences of ORF3a and ORF8 were A39T and I9L, respectively, and had sporadically appeared in one of the viruses. Notably, there were eight amino acids deleted at positions 6–13 in the E protein of two viruses passaged in Caco-2 cells. This deletion was not found in any other viruses. An H125Y mutation in the M protein was found in one or two viruses in A549, Caco-2, and HRT-18 cells, while a T175M M protein mutation was found in one virus passaged in Caco-2 cells. In the translated ORF6 and ORF7a peptides, there were V5F and L86M mutations, respectively, in two viruses passaged in Caco-2 cells and in one virus passaged in HRT-18 cells. Selected mutations in the N protein (S193del, S194T, P207Q, A208T, and R209K) were found in two viruses passaged in Caco-2 cells.

### 2.5 Single nucleotide polymorphisms (SNPs) in the SARS-CoV-2 spike protein after the first passage

**Fig. 4:**
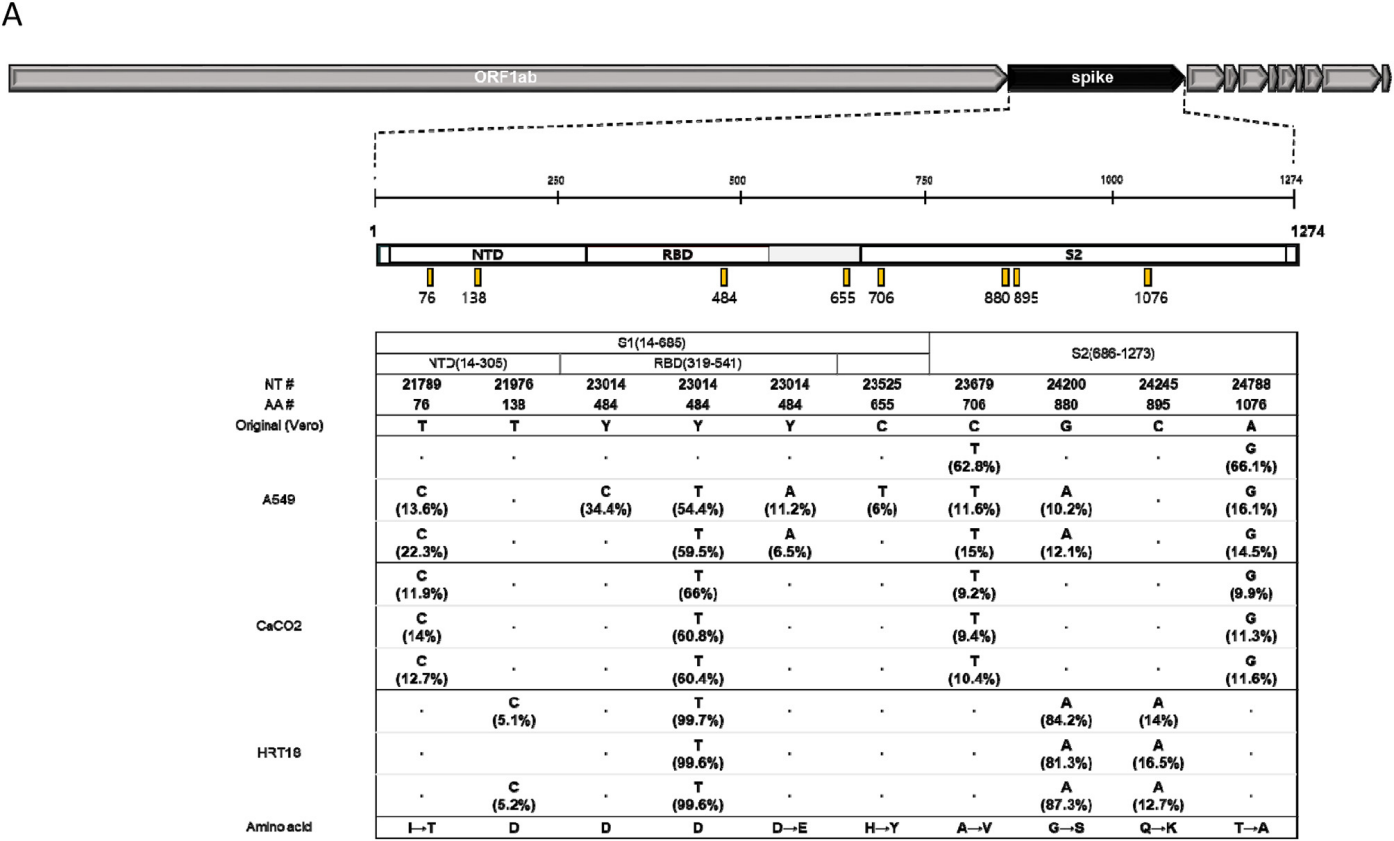

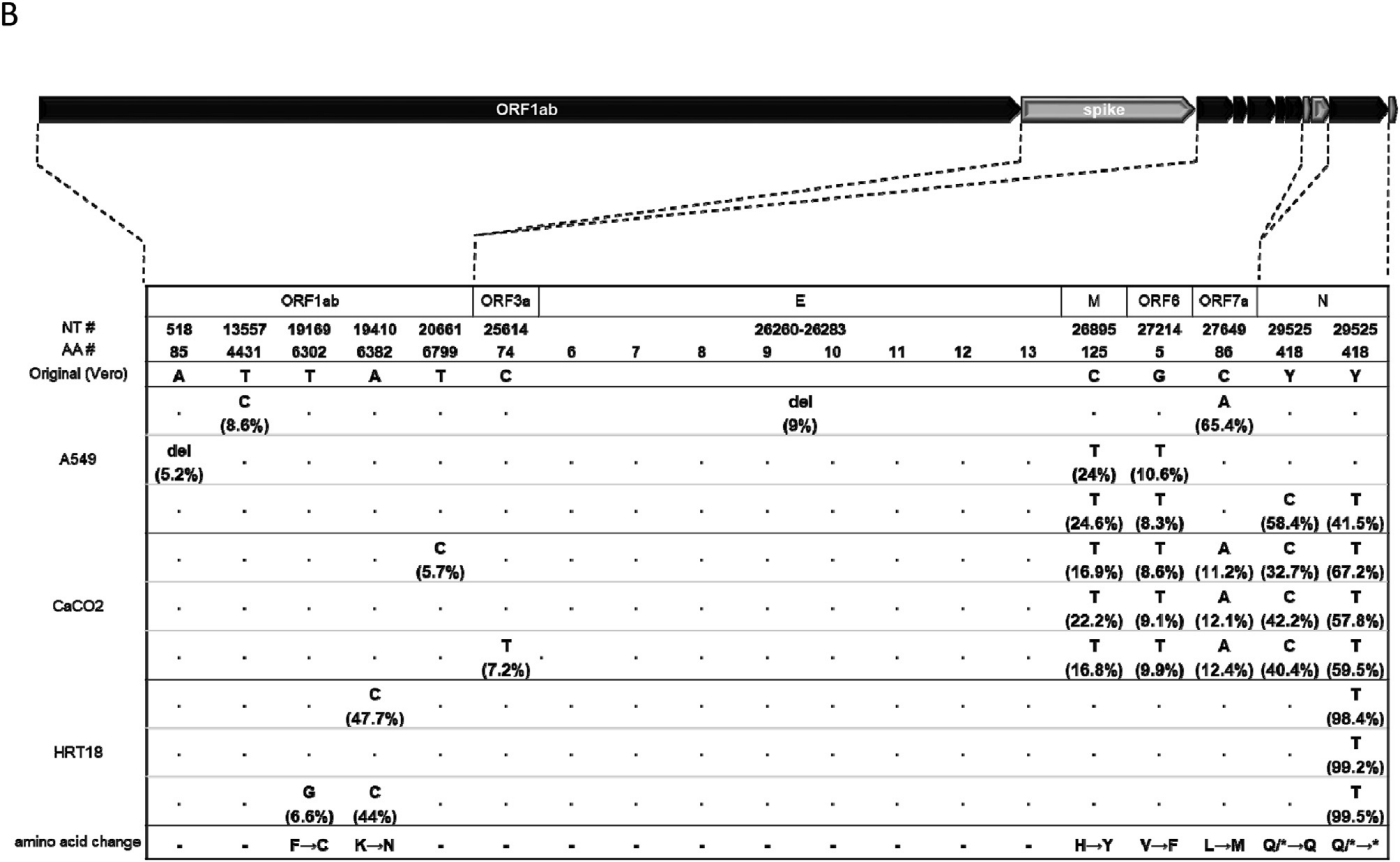
SNPs found in SARS-CoV-2 after one passage in human cells. Consensus genomic sequence of the original SARS-CoV-2 isolate was used as a reference for SNP analysis. A, SNPs on spike protein genes. B, SNPs on all other genes. Nucleotide position number (NT#) was annotated from a reference strain of SARS-CoV-2 (Wuhan-Hu-1, GenBank accession number NC_045512). Variant frequency at each SNP is presented as % value. The resulting synonymous or non-synonymous amino acid substitutions are presented in the last row.

The raw data for SNPs analysis is presented in supplement tables 3, 4, and 5. A total of eight SNPs were found in the spike proteins of SARS-CoV-2 isolates after the first passage through each cell line (Fig. 4a). An SNP at position 21,789 (amino acid 76, NTD domain) was found in all viruses from Caco-2 cells and in two viruses from A549 cells. The original nucleotide at this position was thymine (T), but 11.9–22.3% of the sequenced viruses mutated to cytosine (C), which results in a non-synonymous substitution of isoleucine to threonine. An SNP at position 21,976 (amino acid 138, NTD domain) was observed in two viruses from HRT-18 cells (T21,976C, 5.1–5.2% variant frequency) but resulted in a synonymous substitution of amino acids. In the RBD, the only SNP after one passage was at position 23,014. Although the original SARS-CoV-2 already had C or T nucleotides at the position, we found a higher variant frequency of T (54.5–99.6%) in viruses after one passage. This SNP did not result in an amino acid change at this position. However, an adenine substitution at the same position was found in two viruses from A549 cells (variant frequency of 6.5–11.2%), resulting in a non-synonymous substitution from aspartate to glutamate. In addition, a C23,525T (H655Y) substitution was observed in one virus after the first passage in A549 cells.

In the S2 domain, four SNPs resulting in non-synonymous substitutions were found. C23,679T (A706V) and A24,788G (T1076A) were found in all viruses from the first passage in A549 and Caco-2 cells. In contrast, C24,245A (Q895K) was observed only in viruses from the first passage in HRT-18 cells. G24,200A (G880S) was found in two viruses from the first passage in A549 cells and in all viruses from HRT-18 cells. However, the variant frequency was higher in HRT-18 cells (81.3–84.2%) than in A549 cells (10.2–12.1%).

### 2.6 Additional SNPs in SARS-CoV-2 after the first passage

Overall, ORF1ab had five SNPs that were found in one or two viruses per site (Fig. 4b). Among them, T19,169G (F6302C) and A19,410C (K6382N) resulted in non-synonymous substitutions and were found only in viruses from the first passage in HRT-18 cells. Deletion of nucleotides 26,260–26,283, which corresponds to the eight-amino-acid deletion in the E protein, was analyzed as an SNP with a 9% variant frequency in one virus from the first passage in A549 cells. There was one SNP each causing a non-synonymous substitutions in the M protein, ORF6, and ORF7a, observed in viruses from A549 and Caco-2 cells. However, no SNPs in these genes were found in viruses from the first passage in HRT-18 cells. C26,895T (H125Y) in the M protein gene and G27,214T (V5F) in ORF6 were found in all viruses from the first passage in Caco-2 cells and in two viruses from A549 cells, while C27,649A (L86M) was found in all viruses from the first passage in Caco-2 cells and in one virus from A549 cells. In the case of the N protein, the original SARS-CoV-2 already had C or T nucleotides at position 29,525, which results in either a glutamine residue or termination of translation at amino acid position 418. This variation was also observed in viruses from the first passage in all cell lines. Notably, the T variant was found most frequently (98.4–99.5%) in viruses from HRT-18 cells after one passage.

### 2.7 Comparison of mutations developed during serial passage of SARS-CoV-2

**Fig. 5:**
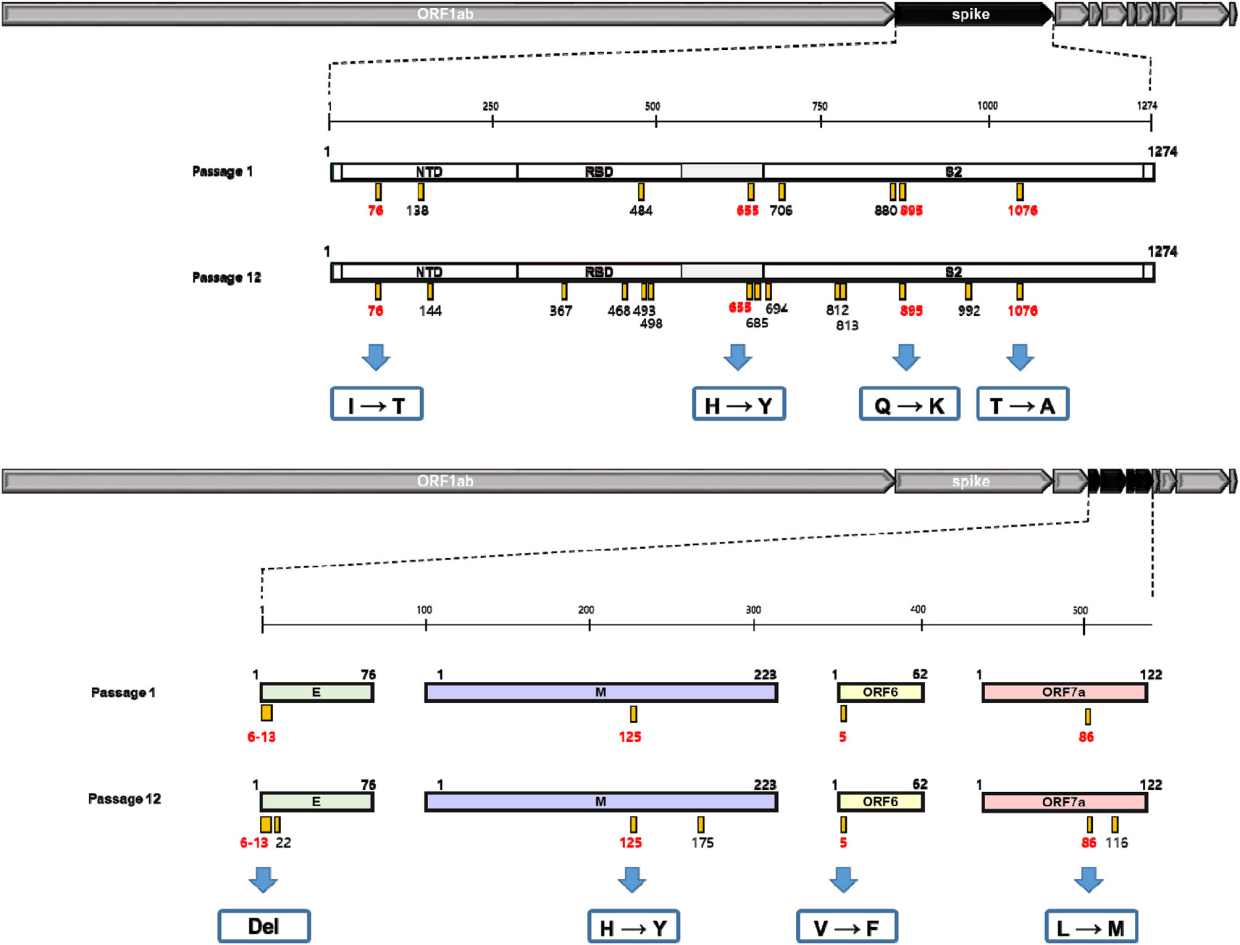
Selected mutations in SARS-CoV-2 after passage 12 are consistent with SNPs in viruses after passage 1 in A549, Caco-2, and HRT-18 cells. Red colored positions represent the selected mutation sites consistent with SNP sites on spike genes (upper) and all other genes (lower); del, deletion of amino acid. (For interpretation of the references to colour in this figure legend, the reader is referred to the web version of this article.)

While considering the selected mutations acquired by SARS-CoV-2 in this study, we found that some SNPs identified after the first passage in each cell line matched with selected mutations after passage 12 (Fig. 5). For the spike protein, selected mutations sites from the passage 12 viruses (I76T, H655Y, Q895K, and T1076A) corresponded with SNP sites found in SARS-CoV-2 after passage 1 in A549, Caco-2, and HRT-18 cells. In addition, eight amino acids deleted at positions 6–13 of E protein in passage 12 viruses from Caco-2 cells were also deleted in viruses from A549 cells after one passage. Several additional substitutions (H125Y in the M protein, V5F in the ORF6 peptide, and L86M in the ORF7a peptide) found in the passage 12 viruses also matched SNPs identified after the first passage.

## 3 Discussion

Since the first outbreak of COVID-19, the causative virus, SARS-CoV-2, has evolved into diverse variants. Following the noticeable increase in the D614G mutation in April 2020, the Omicron variant has acquired over 30 mutations, including those found in the Alpha, Beta, Gamma, and Delta variants [16], [17]. The emergence of SARS-CoV-2 variants continues to affect pathogenicity and vaccine efficacy [18], making it difficult to control COVID-19 worldwide. In a pandemic situation, it is important to track and predict viral mutations to prepare rapid response strategies for variant viruses. With the global effort to track the evolution and transmission of SARS-CoV-2, a considerable amount of sequencing data has been accumulated [19]. Here, we took advantage of the breadth of data available to compare mutations acquired during serial passage to those found in natural SARS-CoV-2 variants.

We subjected an early isolate of SARS-CoV-2 (SARS-CoV-2/human/KOR/KCDC03-NCCP43326/2020) to serial passage over 12 cycles in human cell lines (A549, Caco-2, and HRT-18) in triplicate. Consensus genomic sequences of the passaged viruses were compared to the original SARS-CoV-2 isolate. Although some of the selected mutations found after passaging differed from virus to virus, common mutations were also identified. An E484D substitution on the spike protein was found in all viruses, including the original isolate used here, compared to the SARS-CoV-2 reference genome (Wuhan-Hu-1, GenBank Accession no. NC_045512). This mutation is known to make the virus viable in A549 cells, which are typically non-permissive for SARS-CoV-2 culture [20]. Here, we used an early isolate of SARS-CoV-2 prepared from four passages in Vero cells. The E484D mutation may have been generated in the passaging process, allowing replication in the A549 cells in this study.

Host cell-specific mutations on the spike protein were not common (Fig. 3a). Rather, the same selected mutations were found regardless of which host cell was used for passaging. As the host cells in this study were of human origin, the selected spike protein mutations may be associated with host species-related selection pressure. Notably, several selected mutations after serial passage in A549, Caco-2, and HRT-18 cells were consistent with mutations found in recent wild-type variants of SARS-CoV-2. For example, Q493R and Q498R substitutions in the RBD were identified in this study and are consistent with key amino acid substitutions in the Omicron variant, which was first reported on November 24, 2021, in South Africa [21]. These selected mutations in the spike protein are also associated with mutations in mouse-adapted SARS-CoV-2 (Q493K and Q498H) [22]. In addition, the Y144del and H655Y substitutions reported in this study have naturally arisen in the B.1.1.7 (Alpha), P.1 (Gamma), and B.1.1.529 (Omicron) variants [17], [23]. Thus, serial passage of an early SARS-CoV-2 isolate in human cell lines revealed several selected mutations in the spike protein that are consistent with mutations in recent natural variants. This indicates that there may be a predictable pattern in the selected mutations of SARS-CoV-2 during its evolution in human cells. In addition, as the sequence reads of SARS-CoV-2 in this study might be originated from the viral genome, intermediate antigenome, and subgenomic transcripts, further study about the main origin of those selected mutations will be helpful to understand the SARS-CoV-2 evolution mechanism.

When analyzing non-spike protein genes, selected mutations were more frequent in viruses passaged in Caco-2 and HRT-18 cells than in those passaged in A549 cells (Fig. 3b). V5F and L86M substitutions in ORF6 and ORF7a were not observed in viruses passaged in A549 cells. An eight-amino-acid deletion in the E protein and a five-amino-acid substitution in the N protein were only observed in viruses passaged in Caco-2 cells. Importantly, the Caco-2 and HRT-18 cell lines both originate from human colorectal adenocarcinomas, whereas the A549 cell line was derived from lung carcinoma [24]. Based on the observed divergence in mutation occurrence, these viral genes may be affected by host cell type, even within the same host species. Considering the existing evidence that SARS-CoV-2 infection can cause gastrointestinal symptoms [25], additional studies are needed to understand the possible relationship between the colorectal adenocarcinoma cell line-specific mutations found in this study, gastrointestinal infections, and viral adaptation.

We also analyzed SNPs after the first passage of the original SARS-CoV-2 in A549, Caco-2, and HRT-18 cells (Fig. 4a and 4b). We hypothesized that, when facing new host cell environments, some mutations were tentatively selected as SNPs during viral replication. In the spike protein, nucleotide position 23,014 (corresponding to amino acid 484) represented an SNP with C, T, and A nucleotide substitutions. Although this site was ultimately selected as aspartate (D) after 12 passages in all cell types, it may be crucial for viral adaptation in host cells; importantly, E484K and E484D have been associated with a protective immune response and viral permissiveness in several host cells [9], [10], [11], [20]. A variety of SNPs were identified in non-spike protein genes, but most of them were not maintained over 12 passages in each cell line; in general, SNPs may be generated or disappear throughout serial passaging. However, eight SNPs were identified after the first passage and continuously found after 12 passages in each cell line. These SNPs resulted in non-synonymous amino acid substitutions (I76T, H655Y, Q895K, and T1076A on the spike protein; H125Y on the M protein; V5F on the ORF6 protein; L86M on the ORF7a protein; and an eight-amino-acid deletion in the E protein). These findings indicate that SARS-CoV-2 undergoes selective pressure in new host cells immediately following infection, impacting future viral progeny.

In conclusion, serial passage of an early SARS-CoV-2 isolate in three types of human-derived cell lines confirmed that selected mutations arising from *in vitro* passaging match those found in recent natural SARS-CoV-2 variants. In addition, we found that several SNPs identified after one round of passaging are consistently observed as mutation sites in serially passaged viruses. This indicates that there may be unique mutations that occur early in SARS-CoV-2 infection depending on the host cell environment. However, in this experiment, key natural mutations such as D614G were not observed, which could be due to the specific host cells used or the *in vitro* culture system. Further studies on the mutation patterns of SARS-CoV-2 in diverse host environments, such as under varying immune and antiviral pressures [26], [27], will help predict mutations in future SARS-CoV-2 variants.

## 4 Materials and methods

### 4.1 Virus and cells

The SARS-CoV-2 isolate (SARS-CoV-2/human/KOR/KCDC03-NCCP43326/2020, GenBank accession No. MW466791 in 2020, Korea) was kindly provided by the National Culture Collection for Pathogens (NCCP) (Cheongju, Korea). The virus was passaged four times in Vero cells and used for the experiments in this study.

Vero, A549, Caco-2, and HRT-18 cell lines were used for the SARS-CoV-2 passages. Vero cells were cultured in Dulbecco’s modified Eagle’s medium (DMEM; Welgene, Daegu, Korea, Cat. LM 001-07) supplemented with 2% fetal bovine serum (FBS; Gibco, NY, USA, CAT. 16000044) and 1% penicillin–streptomycin (10,000 IU/ml each) (Gibco, NY, United States, CAT. 15140122) at 37 °C in a 5% CO_2_ incubator. Caco-2 cells were cultured in minimal essential medium (MEM; Welgene Inc., Daegu, Korea, CAT. LM 007-01) supplemented with 2% FBS and 1% penicillin–streptomycin (10,000 IU/ml each). For A549 and HRT-18 cells, RPMI 1640 (Welgene Inc., Daegu, Korea, CAT. LM 001-01) supplemented with 2% FBS and 1% penicillin–streptomycin (10,000 IU/ml each) was used.

All experiments involving the SARS-CoV-2 isolate were performed at the Animal Biosafety Level 3 (ABL-3) facility of the Korea National Primate Research Center at the Korea Research Institute of Bioscience and Biotechnology, Korea (permission number KRIBB-IBC-20200206).

### 4.2 SARS-CoV-2 serial passages in A549, Caco-2, and HRT-18 cell lines

The experimental stock of the SARS-CoV-2 isolate was prepared from the virus passaged 4 times in Vero cells at a titer of 8 × 10^4^ plaque-forming units (PFUs)/ml. One milliliter of the prepared stock virus was inoculated into a monolayer of A549, Caco-2, and HRT-18 cell lines in a 75T flask for 1 h at 37 °C in a 5% CO_2_ incubator with gentle shaking at 15-min intervals. Next, the monolayers were washed twice with DPBS (Welgene Inc., Daegu, Korea, CAT. LB 001-02) and incubated with fresh maintenance medium, as described above, for 3 days. The virus inoculation was performed in triplicate for each cell line (Fig. 1).

The cultured cell supernatants of the virus-infected A549, Caco-2, and HRT-18 cells were centrifuged at 3000×*g* for 10 min for use in the next passage and stored at −80 °C. In the first passage, the cells remaining after supernatant collection were detached via trypsin treatment and centrifuged at 3000 × *g* for 10 min. The cell pellets were then resuspended to a volume of 250 µl with MEM or RPMI, depending on the cultured cells, to extract RNA for high-throughput sequencing.

For serial passages, 1 ml of the centrifuged supernatant was inoculated into a monolayer of A549, Caco-2, and HRT-18 cell lines in a 75T flask for 1 h at 37 °C in a 5% CO_2_ incubator with gentle shaking at 15-min intervals. Next, the monolayers were washed twice with DPBS and incubated with fresh maintenance medium for 3 days. The serial passage of SARS-CoV-2 in A549, Caco-2, and HRT-18 cell lines was continued until passage 12. The cultured cell supernatants of the virus-infected A549, Caco-2, and HRT-18 cells in passages 1, 4, 8, and 12 were centrifuged at 3000×*g* for 10 min and used to extract RNA for high-throughput sequencing.

### 4.3 RNA extraction and high-throughput sequencing

RNA was extracted from the original SARS-CoV-2 strain obtained from the Vero cell culture supernatants of the virus-infected A549, Caco-2, and HRT-18 cells at passage 1, 4, 8, and 12 using the QIAamp Viral RNA Mini Kit (Qiagen, Germany, CAT. 52904), following the manufacturer’s instructions. While all the RNA extracts were used for the SARS-CoV-2 real-time RT-PCR, RNA from the passage 12 samples were further used for high-throughput sequencing. In addition, RNA was extracted from the cells with the first passage of the SARS-CoV-2 isolate using TRIzol LS Reagent (Thermo Fisher Scientific Inc., USA, CAT. 10296028), following the manufacturer’s manual, to study the early mutation pattern of the virus through high-throughput sequencing.

Twenty-one RNA samples were submitted to Macrogen, Inc. (Seoul, Korea) for high-throughput sequencing. Briefly, total cDNA libraries were prepared from the extracted RNA using the Illumina TruSeq Strand Total RNA LT kit and sequenced using the Illumina NovaSeq6000 (output:5Gb, 100PE) platform according to the manufacturer’s instructions. The library QC data is in supplement table 1. The sequencing data of total reads, trimmed reads, and mapped reads with coverage data were calculated for each experimental group as shown in supplement table 2. All raw sequence data were submitted and deposited in the Sequence Read Archive database under SRA accession number SRP349622.

### 4.4 SARS-CoV-2 genomic sequence and mutation analysis

Two separate fastq files per sample were imported as paired-sequence reads with an insert size of 200 to Geneious Prime 2021.2.2. The raw reads were trimmed using BBDuk adaptor and quality trimming version 38.84 [28]. The trimmed reads of the original SARS-CoV-2 isolate in Vero cells and SARS-CoV-2 passaged 12 times in A549, Caco-2, and HRT-18 were mapped to the sequence of the SARS-CoV-2 isolate Wuhan-Hu-1 (GenBank accession number. NC_045512) using the Geneious Prime Bowtie 2 plugin version 2.3.0. [29]. Three consensus genomic sequences in the same group were obtained from mapped data and aligned using the MAFFT v7.450 alignment auto algorithm [30]. In addition, all consensus genomic sequences were annotated and analyzed using Geneious software. The selected mutations were analyzed with the consensus genomic sequences of SARS-CoV-2 passaged 12 times in A549, Caco-2, and HRT-18 cells compared to those of the original SARS-CoV-12 isolates in Vero cells.

For single nucleotide polymorphism (SNP) analysis, the raw reads of the first-passaged SARS-CoV-2 on A549, Caco-2, and HRT-18 cells were imported and trimmed using the same methods as described above. The trimmed reads were mapped to the consensus genomic sequence obtained from the original SARS-CoV-2 isolates using Bowtie 2. Consensus sequences of the original SARS-CoV-2 was generated with the mapped reads based on the highest quality threshold of 60% (Bases matching at least 60% of total adjusted chromatogram quality) as recommended in the Geneious software. The mean read depth of coverages for the consensus genomic sequences of the original SARS-CoV-2 were 11,073, 100,488, and 54,372 in triplicate experiments (supplement table 2). After reads mapping to the consensus genomic sequences, SNPs and INDELs were found with a 0.05 minimum variant frequency with a 10^-6^ maximum variant p-value and 10^-5^ minimum strand-bias p-value (when exceeding 65% bias) based on the Geneious software workflow, “Map reads then find variations/SNPs”. The coverage and variant frequency data of the SNPs and INDELs in each experimental group were presented in supplement tables 3, 4, and 5. Synonymous and non-synonymous substitutions of amino acids at the SNPs were also analyzed.

### 4.5 Real-time RT-PCR

RNAs extracted from the culture supernatants of the virus-infected A549, Caco-2, and HRT-18 cells in passages 1, 4, 8, and 12 were tested in duplicate with real-time RT-PCR using the primers and probe targeting the ORF1b region of the virus [31]. Primer and probe sequences were 5′-TGGGGYTTTACRGGTAACCT-3′ (Forward: Y = C/T, R = A/G), 5′-AACRCGCTTAACAAAGCACTC-3′ (reverse, R = A/G), and 5-TAGTTGTGATGCWATCATGACTAG-3 (probe in 5′-FAM/ZEN/3′-IBFQ format; W = A/T).

To quantitatively measure the viral RNA copy numbers, SARS-CoV-2 RNA standard (Twist Synthetic SARS-CoV-2 RNA Control (MT007544.1, Twist Bioscience, USA) was serially diluted 10-fold with RNase-free distilled water to prepare RNA copy numbers between 2,000,000 and 20 copies/reaction. Real-time RT-PCR was performed using the QiantiTect Probe RT-PCR Kit (Qiagen, Germany). 204443), according to the manufacturer’s instructions. Briefly, 2 µl of RNA template, 0.8 µl of each primer (10 pmol/µl), 0.4 µl of probe (10 pmol/µl), 10 µl of 2x QuantiTect probe RT-PCR Master Mix, 0.2 µl of QuantiTect RT Mix, and 5.8 µl of RNase-free water were mixed to 20 µl for the reaction. The thermal conditions were 50 °C for 30 min, 95 °C for 15 min, followed by 40 cycles of 94 °C for 15 s and 60 °C for 60 s.

## 5 Data availability

The genome sequence of the SARS-CoV-2 isolate used in this study (SARS-CoV-2/human/KOR/KCDC03-NCCP43326/2020) has been deposited in GenBank (accession number MW466791). RNA-sequencing data has been deposited in the Gene Expression Omnibus database (GEO Series accession number GSE190350). Raw sequence reads are available under the BioProject accession number PRJNA786802 with SRA accession number SRP349622.

### CRediT authorship contribution statement

**Hoyin Chung:** Conceptualization, Formal analysis, Investigation, Writing – original draft. **Ji Yeong Noh:** Methodology, Formal analysis, Data curation, Writing – original draft. **Bon-Sang Koo:** Formal analysis, Resources. **Jung Joo Hong:** Conceptualization, Formal analysis, Resources, Writing – review & editing, Supervision. **Hye Kwon Kim:** Conceptualization, Methodology, Formal analysis, Writing – original draft, Writing – review & editing, Supervision.

## Declaration of Competing Interest

The authors declare that they have no known competing financial interests or personal relationships that could have appeared to influence the work reported in this paper.
